# Supplementary material for: Clinical Manifestations and Cytokine Profiles of the Th1, Th2, and Th17 Response Associated with SARS-CoV-2 Omicron Subvariants
Source: Biomedicines. 2025 Aug 31;13(9):2128. doi: 10.3390/biomedicines13092128 (PMC12467591; doi:10.3390/biomedicines13092128)
Supplement: Supplementary file 1 [file biomedicines-13-02128-s001.zip › biomedicines-3792841-supplementary.pdf]

Supplementary material for the article entitled:

Clinical Manifestations and Cytokine profiles of the Th1, Th2 and Th17 response Associated with SARS-CoV-2 Omicron Subvariants

Journal: Biomedicines [EISSN 2227-9059]

Table S1. Statistical Associations between Reported Symptoms and Cytokine Concentrations in COVID-19 Positive Patients.

Symptoms	Cytokines (p value [a])						
(n = 40)	IL-17A	IFN-γ	TNF	IL-10	IL-6	IL-2	IL-4
Pharyngitis	0.590	0.143	0.200	0.292	0.334	0.158	0.326
Cough	0.219	0.468	0.558	0.876	0.651	0.100	0.975
Fever	0.797	0.349	0.785	0.290	0.471	0.365	0.645
Nasal discharge	0.995	0.992	0.990	1.000	0.990	0.990	0.990
Fatigue	0.514	0.278	0.121	0.643	0.680	0.093	0.677
Headache	0.130	0.961	0.679	0.690	0.098	0.447	0.401
Joint pain	0.372	0.684	0.845	0.693	0.285	0.908	0.882
Muscle pain	0.712	0.767	0.243	0.314	0.372	0.996	0.402
Painful breathing	0.275	0.104	0.915	0.226	0.759	1.000	0.258
Dyspnea	0.684	0.312	0.480	0.545	0.660	0.300	0.625
Diarrhea	0.856	0.378	0.870	0.686	0.590	0.978	0.755
Chills	0.626	0.467	0.154	0.184	0.270	0.446	0.378
Abdominal pain	0.634	0.377	0.026	0.254	0.324	0.670	0.936
Loss of taste	0.915	0.038	0.997	0.165	0.156	0.332	0.723
Loss of smell	0.853	0.255	0.904	0.726	0.747	0.687	0.631
Emesis	0.579	0.854	0.176	0.734	0.227	0.209	0.729

n = number of individuals; [a] Binomial logistic regression test.

Table S2. P values after correction for statistical associations between reported symptoms and cytokine concentrations in COVID-19-positive patients.

Symptoms	Cytokines (p value [a])						
(n = 40)	IL-17A	IFN-γ	TNF	IL-10	IL-6	IL-2	IL-4
Pharyngitis	1.000	0.962	0.962	0.962	0.962	0.962	0.962
Cough	0.962	1.000	1.000	1.000	1.000	0.962	1.000
Fever	1.000	0.962	1.000	0.962	1.000	0.962	1.000
Nasal discharge	1.000	1.000	1.000	1.000	1.000	1.000	1.000
Fatigue	1.000	0.962	0.962	1.000	1.000	0.962	1.000
Headache	1.000	0.962	1.000	1.000	0.962	1.000	1.000
Joint pain	1.000	1.000	1.000	1.000	1.000	1.000	1.000
Muscle pain	1.000	1.000	0.962	0.962	1.000	1.000	1.000
Painful breathing	0.962	0.962	1.000	0.962	1.000	1.000	0.962
Dyspnea	1.000	0.962	1.000	1.000	1.000	0.962	1.000
Diarrhea	1.000	1.000	1.000	1.000	1.000	1.000	1.000
Chills	1.000	1.000	0.962	0.962	1.000	1.000	1.000
Abdominal pain	1.000	1.000	0.962	1.000	1.000	1.000	1.000
Loss of taste	1.000	0.962	1.000	0.962	0.962	1.000	1.000
Loss of smell	1.000	1.000	1.000	1.000	1.000	1.000	1.000
Emesis	1.000	1.000	0.962	1.000	1.000	1.000	1.000

n = number of individuals; [a] Correction of p values using the Benjamini-Hochberg (BH) method to control the false discovery rate (FDR).
